# A feasibility trial of a digital mindfulness-based intervention to improve asthma-related quality of life for primary care patients with asthma

**DOI:** 10.1007/s10865-021-00249-3

**Published:** 2021-08-27

**Authors:** Ben Ainsworth, Sabina Stanescu, Beth Stuart, Daniel Russell, Megan Liddiard, Ratko Djukanovic, Mike Thomas

**Affiliations:** 1 Department of Psychology, Faculty of Humanities and Social Sciences, University of Bath, Bath, UK; 2 NIHR Biomedical Research Centre, University Hospitals Southampton NHS Foundation Trust, Southampton, Hampshire, UK; 3 School of Psychology, Faculty of Life and Environmental Sciences, University of Southampton, Southampton, UK; 4 Primary Care Research Centre, School of Primary Care, Population Sciences and Medical Education, Faculty of Medicine, University of Southampton, Southampton, UK; 5 Southampton, UK; 6 Clinical and Experimental Sciences, Faculty of Medicine, University of Southampton, Southampton, UK

**Keywords:** Asthma, Mindfulness, Quality of life, Asthma, Primary care, Anxiety

## Abstract

Asthma outcomes remain suboptimal, despite effective pharmacotherapy. Psychological dysfunction (such as anxiety) is common, and associated with poorer outcomes. We evaluated a digital mindfulness programme as an intervention to improve asthma-related quality of life for primary care patients, in a prospectively registered randomized-controlled feasibility study. We offered ‘Headspace’, a widely-used digital mindfulness intervention, to adults with asthma through 16 UK GP practices. Participants were randomized on a 2:1 basis to the mindfulness intervention, or waitlist control. Participants completed questionnaires (including asthma symptom control, asthma-related quality of life, anxiety, depression) at baseline, 6-week and 3-month follow-up. 116 participants completed primary outcomes at 3-month follow-up: intervention 73 (79%), control 43 (84%). Compared to baseline, the intervention group but not the control group reported significantly improved asthma-related quality of life, with a between-group difference favoring the intervention group that was not significant (Mean difference = 0.15, 95%CI − 0.13 to 0.42). Intervention use varied (ranging from 0 to 192 times) but was generally high. Digital mindfulness interventions are feasible and acceptable adjunct treatments for mild and moderate asthma to target quality of life. Further research should adapt ‘generic’ mindfulness-based stress-reduction to maximize effectiveness for asthma, and validate our findings in a fully-powered randomized controlled trial.

*Trial registration* Prospectively registered: ISRCTN52212323.

**Supplementary Information:**

The online version contains supplementary material available at 10.1007/s10865-021-00249-3.

## Introduction

Asthma is a multifaceted chronic disease, with recent estimates that it affects 339 million people of all ages worldwide and 6.5% of the UK population (Bloom et al., 2019). Although evidence suggests that modern pharmacotherapy can achieve good asthma control in clinical trials (Bateman et al., 2004), in reality the heterogenous clinical and behavioral phenotypes mean that asthma outcomes remain suboptimal, and many patients continue to experience persistent symptoms and impaired quality of life (Demoly et al., 2010).

The causes of these suboptimal therapeutic outcomes are complex and wide-ranging, including poor self-management (i.e. corticosteroid inhaler adherence and technique; see Thomas, 2015 for a review). Increasingly the role of psychological comorbidity including anxiety, depression and panic has become apparent (Gada et al., 2014; Goldney et al., 2003; Hasler et al., 2005; Shaw et al., 2015). Anxiety and depression-related psychological dysfunction are up to six times more common in people with an asthma diagnosis (Goodwin et al., 2003; Lavoie et al., 2006), and even more likely with difficult-to-control asthma (Lavoie et al., 2006). With frequent experiences of unpredictable and potentially life-threatening breathlessness, psychological dysfunction is also associated with avoidant coping strategies (leading to lower quality of life; Adams et al., 2004; Cluley & Cochrane, 2001) and increased healthcare utilisation (Richardson et al., 2008). Recent reviews have highlighted the need for appropriate treatment that considers these psychological aspects that will improve patient well-being and asthma control (Baiardini et al., 2015).

Proactive asthma self-management can improve asthma control and reduce healthcare utilisation in a cost-effective manner (Pinnock et al., 2017). As well as promoting appropriate pharmacological management (e.g. regular medication use, maintaining an up-to-date asthma action plan), self-management recommendations for asthma have included non-pharmacological methods such as lifestyle modification, including smoking cessation, weight reduction, breathing retraining (Miles et al., 2017; Bruton et al., 2017). Current research in asthma suggests that psychological interventions that aim to improve asthma-related outcomes can potentially be effective (Yorke et al., 2007). However, evidence for specific treatments generally remains inconclusive due to the low quality and volume of research performed, the variety of interventions investigated (including relaxation, biofeedback, mindfulness and self-management) and the variety of health-related outcome measures reported (Yorke et al., 2015). A recent large-scale randomized controlled trial (RCT) found that self-guided breathing exercises for asthma were effective and cost-effective to improve quality of life (Bruton et al., 2017), and this intervention is now advocated in evidence-based asthma guidelines (James & Lyttle, 2016; Reddel et al., 2015).

Proposed mechanisms behind the purported benefits of non-pharmacological interventions include maladaptive behaviors (such as poor medication adherence or dysfunctional breathing) and cognitive-affective factors (such as inaccurate symptom perception; Janssens et al., 2012), as well as comorbid psychological conditions (such as anxiety and depression). Therefore, psychological interventions that target dysfunctional cognitive-affective mechanisms may benefit asthma quality of life (Janssens et al., 2009).

Mindfulness meditation-based interventions (MBIs), which involve deliberate, non-judgemental attention and acceptance of experiences (Brown & Ryan, 2003), can potentially offer such a benefit for people with asthma. Mindfulness-based stress reduction (MBSR) and mindfulness-based cognitive therapy (MBCT) are common treatments for anxiety and depression (Strauss et al., 2014) and have demonstrated benefit across a range of chronic conditions (e.g. fibromyalgia, cancer, arthritis and cardiovascular disease (see Bohlmeijer et al., 2010). Although, as with other non-pharmacological treatments, evidence for asthma-specific benefits is inconclusive (Paudyal et al., 2018), qualitative interviews with patients taking part in mindfulness-based cognitive therapy found improved body awareness and acceptance of symptoms. Cross-sectional studies have found that higher dispositional mindfulness (i.e. an innate capacity to pay attention with a non-judgemental attitude) is associated with reduced asthma symptoms in individuals with poorly controlled asthma (Kraemer & McLeish, 2019) and college students with asthma (Shi et al., 2018). Adults with mild, moderate or severe asthma who were randomized to an 8-week MBSR course (vs. psychoeducation) showed improved quality of life and perceived stress but no improvement in lung function (Pbert et al., 2012).

A common barrier to the implementation of MBIs in chronic disease is the burden of attending the weekly group sessions—for example, a standard MBSR course might consist of 8 two-hour sessions once per week, with additional self-practice (Ainsworth et al., 2020; Simpson et al., 2018). MBIs are complex behavioral interventions and it is therefore possible that they may require innovative delivery models to maximize access and effectiveness across different patient groups and to achieve cost-effectiveness (Demarzo et al., 2015). Digital mindfulness interventions could potentially offer alternatives to traditional programmes, allowing accessibility to content that has been created and validated by experts, across a heterogenous population at low-cost. Digital self-management support interventions have been successfully trialled in asthma, with patient acceptability (Ainsworth et al, 2019a; Morrison et al., 2016). Web-based MBIs have shown some benefit in alleviating symptom burden across non-respiratory chronic conditions (Toivonen et al., 2017), but to date a digital mindfulness intervention has not been evaluated for adults with asthma.

This study aimed to explore the feasibility of using ‘Headspace’, a market-leading digital mindfulness intervention that is commercially available (Mani et al., 2015) for improving patient-reported outcomes for people with mild and moderate asthma treated in primary care, and to estimate effect size for a subsequent fully powered trial.

## Methods

### Objectives

Specific study objectives were to:

1. Explore recruitment procedures including rates of invitation response, study recruitment, randomization and retention in a feasibility pilot randomized controlled trial of a digital mindfulness intervention for people with asthma.
2. Describe and evaluate changes in of baseline and 3-month follow-up self-report measures of quality of life, asthma control, anxiety and depression.
3. Examine intervention usage and engagement to inform a future modified intervention.

### Design

The study was a prospective randomized-controlled feasibility trial comparing adult primary care patients with asthma who were given free access to the digital mindfulness programme with patients who received their usual asthma management, using simple randomization (weighted: 2 intervention vs. 1 control, in order to examine intervention usage and engagement in depth).

Ethical approval was given by the South Central Hampshire Research Ethics Committee: 17/SC/0088. No formal power calculation was conducted as this was a feasibility study.

### Participants

Participants meeting the inclusion criteria were identified in searches of GP electronic clinical records and invited from practices in Hampshire, UK. Target sample size was 120 (80 intervention, 40 control), considered sufficient to explore the feasibility of our trial procedures and inform future intervention optimisation (Lewis et al., 2021).

Inclusion criteria: Over 18 years old, clinical asthma diagnosis and current treatment in primary care (confirmed by one or more asthma medication prescription in previous year).

Exclusion criteria: previous diagnosis of major or unstable comorbid psychological disorders, other than anxiety or depression, currently participating in another asthma interventional study, acute exacerbation of asthma requiring a course of oral steroids within previous 28 days, asthma treated in secondary care.

Recruitment was conducted from July 2017 to April 2018.

### Outcome measures

The study and measures were prospectively registered on the ISRCTN Registry (reference 52212323). The main outcome of the study was the feasibility of trial procedures (recruitment and randomization rates, intervention engagement, completion and acceptability of outcome measures).

#### Self-report and clinical measures

Alongside feasibility and recruitment measures, the main study outcome of interest was asthma-related quality of life, measured using the mini-Asthma Quality of Life Questionnaire [AQLQ:(Juniper et al., 1992)], a validated 15-item questionnaire in which participants assess their asthma-related wellbeing over the last two weeks. The overall score is the mean of all items (7 = not impaired at all, 1 = severely impaired), with 4 subscales (symptoms, activities, emotion, environment) with a minimum clinically important difference (MCID) for individual patients of 0.5. A higher score equates to better quality of life. Baseline scores demonstrated good internal reliablity (α = 0.92).

Asthma control was measured with the 6-item Asthma Control Questionnaire [ACQ: (Juniper et al., 1999)], with a lower score equating to improved asthma control (α = 0.88). Anxiety and depression were measured with the Hospital Anxiety and Depression Scale [HADS: (Zigmond & Snaith, 1983); anxiety α = 0.87, depression α = 0.84]. Mindfulness was measured with the Philadelphia Mindfulness Scale [PHLMS: (Cardaciotto et al., 2008); awareness α = 0.83; acceptance α = 0.88]. Medication adherence was measured with the Medical Adherence Report Scale—Asthma [MARS-A: (Mora et al., 2011); α = 0.83].

Participants responding to the study invitation completed online consent and confirmed eligibility on the computerized Lifeguide platform (Yardley et al., 2009) before completing baseline demographic and self-report measures of anxiety, depression, mindfulness and medication adherence. The primary outcome (AQLQ) was completed via post (as it was not available for on-line completion), as was the ACQ.

Participants were randomized by the Lifeguide software and those allocated to the intervention were sent intervention access instructions via email and post. Six week and three month follow-up measurements were conducted via post (asthma quality of life, asthma control) and online (anxiety, depression, mindfulness and medication adherence).

Additional exploratory outcome measures of participant enablement, acceptance and action and illness perceptions were also recorded, alongside qualitative interviews, for a detailed process analysis that will be reported in a separate paper (Stanescu et al., 2021).

### Intervention

*Mindfulness Intervention:* The commercially-available Headspace app (‘Meditation and Sleep Made Simple—Headspace’, available from http://www.headspace.com/). In an empirical examination of the quality of 23 commercially-available meditation apps, Headspace was rated the highest based on various criteria including engagement, functionality and information quality (Mani et al., 2015). It is available on iOS and Android smartphones, and desktop computers. Supplementary File 1 describes Headspace according to the template for intervention description and replication (TIDieR; Hoffmann et al., 2014).

While Headspace does contain brief online written information (e.g. ‘the science of meditation’) the primary content is 3, 5, 10 and 20 min long audio-guided meditations. The Headspace app contains more than 200 different courses covering a wide range of topics, from stress and mental health to physical health, job performance, and emotional well-being. Individuals new to mindfulness and meditation can learn the fundamentals by engaging with the three “basics” courses, each consisting of ten guided meditations. Upon randomization to the intervention group (or after completing final follow-up if randomized to the waitlist control group), participants were provided with unique redemption codes providing them with 6 months of free access to the complete Headspace content library. Participants were sent standard instructions on how to activate their Headspace accounts using their free access code via email and post.

### Recruitment, retention and adherence

**Fig. 1:**
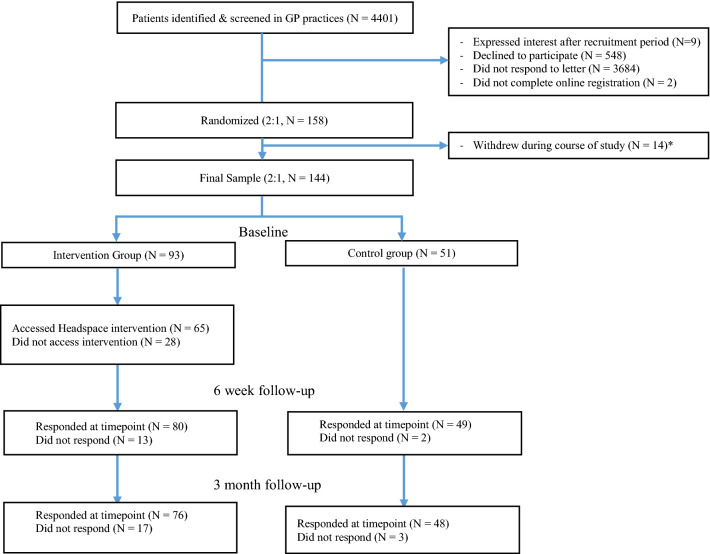
Consort Diagram of recruitment and retention during study. *Note* Participants completed both online and postal measures and are included here if they completed either. A full breakdown of the measures completed by participants at each timepoint (and therefore how many patients were available for subsequent analysis) is available in Table 2. *Reasons for withdrawal were loss of interest (N = 4), lack of time (N = 2), personal issues/illness not related to study (N = 3), wanted to use intervention despite being in control group (N = 1), difficulty using website (N = 1) and no reason given (N = 3)

Participant recruitment was conducted with the NIHR Clinical Research Network (CRN) Wessex, who contacted local GP practices to confirm interest. Staff across 17 GP practices (mean list size 9584) across Hampshire searched patient records, identifying 6243 patients meeting eligibility criteria (see Fig. 1). 4401 patient records were screened by clinicians before being contacted with a letter from their GP and a copy of the participant information sheet. Participants were invited to contact the study team to express interest, or could sign up using the study website. Participants did not receive financial compensation for their involvement.

A total of 158 participants provided informed consent and were randomized after completing baseline questionnaires. Intervention group participants were granted access to the intervention immediately while control group participants were told they would receive access after they had completed further questionnaires in six weeks and three months. 14 participants (5 control, 9 intervention) withdrew during the course of the study leaving a final sample of 144 (control 51 / intervention 93). Reasons for withdrawal are reported in the Consort diagram (Fig. 1). No harms were reported during the study.

548 participants (37% male, age M = 62) returned optional opt-out postal slips with free-text reasons for opting out. Common reasons for opting out included considering asthma as not severe enough (N = 82) or well-controlled (N = 43), no asthma symptoms (N = 20), not having asthma (N = 23), not having access to the internet (N = 82), not interested in meditation (N = 9), already experienced with meditation (N = 12) or too busy to take part (N = 9).

A full CONSORT flow diagram of the study is presented in Fig. 1.

### Analysis

Study outcome data were examined using using SPSS v24 and the results are presented descriptively. Independent group comparisons compared baseline differences. Within-group and between-group changes from baseline in the key outcome measures were assessed, including estimations of proportions reaching the minimum clinically important differences in the AQLQ (MCID: 0.5). The feasibility study was not powered to detect significant differences from pre-test to post-test or between groups, but exploratory comparisons using linear regression, controlling for baseline values of each outcome measure, examined changes in self-report measures between groups to inform future power calculations. Missing data was not imputed and the results presented here represent complete cases only.

## Results

### Participants

Participants had a large range of education, internet experience, meditation experience and time since asthma diagnosis, and were predominantly white. Baseline demographic and outcome measures scores, collected online during study registration, are reported in more detail in Table 1.

**Table 1:** Baseline participant demographic information and outcome scores for intervention and control groups

Measure	Intervention (N = 93)	Control (N = 51)	Mean difference (95% CI)
Age: M (SD)	49.8 (14.7)	53.5 (14.4)	3.7 (− 1.4, 8.8)
Ethnicity (%)	White (97%), Indian (3%),	White (93%), Chinese/South East Asian (2%), Indian (2%), Other (2%)	–
Education (%)	School (22%), Degree/Diploma (57%), Postgraduate (20%), Other (1%)	No formal (5%), School (52%), Degree/Diploma (26%), Postgraduate (14%), other (2%)	–
Weekly internet use: M hours (SD)	17.6 (14.4)	16.4 (14.3)	− 1.3 (− 6.9, 4.3)
Meditation experience (%)	Not heard of it/don’t know about it (14%), never tried it (30%), tried other types of meditation (23%), tried mindfulness (25%), regularly practice mindfulness (7%)	Not heard of it/don’t know about it (24%), never tried it (37%), tried other types of meditation (17%), tried mindfulness (16%), regularly practice mindfulness (5%)	–
Years since diagnosis	28.2 (15.3)	21.1 (16.2)	− 6.8 (− 13.0, − 0.6)*
Asthma-related Quality of Life (AQLQ)	5.32 (1.1)	5.64 (1.0)	0.32 (− 0.05, 0.69)
Symptoms subdomain	5.09 (1.2)	5.52 (1.2)	0.43 (0.01, 0.87)*
Environment subdomain	4.92 (1.4)	5.05 (1.4)	0.13 (− 0.35, 0.62)
Emotions subdomain	5.26 (1.4)	5.76 (1.3)	0.51 (0.02, 0.99)*
Activities subdomain	5.85 (1.1)	6.01 (0.9)	0.17 (− 0.20, 0.54)
Asthma control (ACQ)	1.18 (0.9)	1.06 (0.8)	− 0.12 (− 0.43, 0.18)
Anxiety (HADS-A)	8.24 (4.3)	6.76 (4.1)	− 1.47 (− 2.91, − 0.01)*
Depression (HADS-D)	4.72 (3.9)	3.65 (3.1)	− 1.07 (− 2.33, 0.19)
Mindful awareness (PHLMS-Aw)	31.3 (7.7)	29.6 (8.1)	− 1.74 (− 4.45, 0.97)
Mindful acceptance (PHLMS-Ac)	35.1 (6.8)	33.9 (5.7)	− 1.26 (− 3.46, 0.95)
Medication adherence (MARS-A)	37.9 (8.5)	38.1 (8.4)	0.25 (− 2.68, 3.18)

ACQ (lower scores equate to better control); AQLQ (higher scores equate to greater impairment); HADS (higher scores equate to more anxiety). PHLMS (two subscales of awareness and attention, in which higher scores equate to more mindfulness); MARS-A (higher scores equate to better adherence). (*) denotes group differences in which the 95% CI does not include 0

Baseline group comparisons showed some imbalances between the randomization groups: people randomized to the intervention group were diagnosed longer ago, and had greater impairment on AQLQ symptom and emotion subdomain, higher anxiety scores, greater overall AQLQ impairment and lower asthma control scores. Our primary analysis of follow-up comparisons therefore controlled for baseline differences in each measure by including them as covariates within the regression model.

Baseline comparisons of demographic indicators (calculated with chi-squared comparison) and questionnaire measures (calculated with independent t-tests) between participants who completed the study, and those who dropped out, or did not provide follow-up measures at either 6 weeks or 3 months, showed no differences between groups (see Supplementary File 2).

### Intervention engagement

65 participants in the intervention group (70%) accessed the app through the access code provided on one or more occasions, with a total of 2277 recorded individual sessions. Usage after the 3 month trial period had ended was not included in analysis.

Participants in the intervention group accessed the intervention between 0 and 192 times each (Median 9.0, IQR 0–38.5). 28 participants did not access the intervention at all. Average session length was 7.2 min (SD 3.0) with 682 different practice recordings used across 232 session types. The mean number of days between the first and last use (during the trial) was 51 days.

Participants most frequently accessed the initial introductory session (‘Basics’, Median 6.6 times accessed, Range 13, accessed by 100% of users) as well as ‘Managing Anxiety’ (M 4.0, R 31, 31% user access), Basics 2 (M 3.0, R 13, 55%) and Basics 3 (M 2.6, R 11, 33%). Only the most popular sessions (introductory and anxiety management) were used consistently across participants; most other sessions (eg. stress, self-esteem, sleep) were used by fewer than 5 individuals.

Exploratory analysis allocated participants into users who were non-engagers (0 log ins, N = 26), low engagers (1–9 sessions, N = 26), moderate engagers (10–49 sessions, N = 23) and high engagers (> 50 sessions, N = 18). Baseline group comparisons found no differences in baseline scores of asthma control (F_(3,80)_ = 0.51, *p* = 0.68), asthma-related quality of life (F_(3,80)_ = 0.66, *p* = 0.58), depression (F_(3,89)_ = 1.75, *p* = 0.16), anxiety (F_(3,89)_ = 2.26, *p* = 0.09), mindfulness (F_(3,89)_ = 1.69, *p* = 0.18) or medication adherence (F_(3,89)_ = 1.15, *p* = 0.33).

### Outcome measure response rate.

Total responses to postal and online questionnaire measures are reported in a consort diagram in Fig. 1, and specific outcome measure responses are reported in Table 2. Most participants completed both online and postal questionnaires, with similar completion in both groups. Completion rates were slightly higher in the control group.

**Table 2:** Overall questionnaire response rates

	Intervention N (%) (randomized N = 93)			Control N (%) (randomized N = 51)		
	Baseline	6-week	3-month	Baseline	6-week	3-month
*Questionnaire measures*						
Asthma Quality of Life (AQLQ)	84 (90)	65 (70)	73 (79)	48 (94)	46 (90)	43 (84)
Asthma Control (ACQ)	84 (90)	64 (69)	73 (79)	48 (94)	46 (90)	43 (84)
Anxiety (HADSA)	100 (100)	41 (80)	65 (70)	100 (100)	65 (70)	43 (84)
Depression (HADSD)	100 (100)	41 (80)	65 (70)	100 (100)	65 (70)	43 (84)
Mindful Awareness (PHLMS-Aw)	100 (100)	41 (80)	65 (70)	100 (100)	65 (70)	43 (84)
Mindful Acceptance (PHLMS-Ac)	100 (100)	41 (80)	65 (70)	100 (100)	65 (70)	43 (84)
Medication Adherence (MARS-A)	100 (100)	41 (80)	65 (70)	100 (100)	65 (70)	43 (84)
*Demographic measures*						
Age	91 (98)			50 (98)		
Ethnicity	69 (74)			41 (80)		
Education	69 (74)			41 (80)		
Weekly Internet Use	68 (73)			41 (80)		
Meditation Experience	69 (74)			40 (78)		
*Response type*						
Both postal and online (all measures)	84 (90)	50 (54)	62 (67)	48 (94)	38 (75)	38 (75)
Postal only (AQLQ, ACQ)	0 (0)	15 (16)	11 (12)	0 (0)	8 (16)	5 (10)
Online Only (HADS, PHLMS, MARS-A)	9 (10)	15 (16)	3 (3)	3 (6)	3 (6)	5 (10)
None	0 (0)	13 (14)	17 (18)	0 (0)	2 (4)	3 (6)

Participants who actively withdrew from study (N = 14: 5 control, 9 intervention) were excluded from this analysis, those lost to follow-up were not

Further analyses at each time point included all participants who completed data for that timepoint (for example, participants who completed measures at 3-months but not 6-weeks were still included in the 3-month followup analysis).

### Primary analysis: Asthma Quality of Life Questionnaire and Asthma Control Questionnaire

Follow-up scores are reported in Table 3, with scores for each measure at each time-point, and group comparisons of estimated marginal means (i.e. between-group comparisons corrected for baseline differences, reported in Table 3) at 6-week and 3-month. Between group comparisons were followed by comparisons within each group (baseline vs. follow-up), reported in Table 4.

**Table 3:** Baseline, 6-week and 3-month follow-up questionnaire scores of randomized intervention (N = 93) vs. control participants (N = 51)

	Intervention (M, SD)			Control (M, SD)			Intervention vs. Control Comparison (M, 95% CI)	
Postal measures	Baseline (N = 84)	6-week (N = 64)	3-month (N = 73)	Baseline (N = 48)	6-week (N = 46)	3-month (N = 43)	Baseline vs. 6-week (N = 63 vs 45)	Baseline vs. 3-month (N = 71 vs 42)
Asthma-related Quality of Life (AQLQ)	5.32 (1.1)	5.67 (1.0)	5.77 (0.9)	5.64 (1.0)	5.72 (1.0)	5.77 (0.9)	0.20 (− 0.06, 0.46)	0.15 (− 0.13, 0.42)
Symptoms subdomain	5.09 (1.2)	5.56 (1.1)	5.60 (1.1)	5.52 (1.2)	5.60 (1.1)	5.60 (1.1)	0.25 (− 0.08, 0.59)	0.15 (− 0.21, 0.51)
Environment subdomain	4.92 (1.4)	5.33 (1.1)	5.58 (1.2)	5.05 (1.3)	5.30 (1.4)	5.58 (1.3)	0.10 (− 0.24, 0.45)	0.26 (− 0.08, 0.60)
Emotions subdomain	5.26 (1.4)	5.57 (1.4)	5.74 (1.2)	5.76 (1.3)	5.86 (1.3)	5.74 (1.1)	0.08 (− 0.32, 0.49)	− 0.11 (− 0.45, 0.23)
Activities subdomain	5.85 (1.1)	6.15 (1.0)	6.14 (1.0)	6.02 (0.9)	6.07 (1.1)	6.14 (1.0)	0.20 (− 0.09, 0.48)	0.20 (− 0.11, 0.51)
Asthma control (ACQ)	1.18 (0.9)	1.02 (0.9)	1.00 (0.8)	1.08 (0.8)	1.14 (0.8)	1.15 (0.9)	− 0.26 (− 0.49, − 0.03)*	− 0.17 (-0.44, 0.10)

Online measures	Baseline (N = 93)	6-week (N = 65)	3-month (N = 65)	Baseline (N = 51)	6-week (N = 41)	3-month (N = 43)	Baseline vs. 6-week (N = 65 vs. 41)	Baseline vs. 3-month (N = 65 vs. 43)
Anxiety (HADS-A)	8.24 (4.3)	8.58 (3.3)	7.46 (3.5)	6.76 (4.1)	7.78 (2.8)	7.40 (3.0)	− 0.18 (− 1.00, 0.63)	− 0.62 (− 1.50, 0.27)
Depression (HADS-D)	4.72 (3.9)	3.86 (3.6)	3.49 (3.3)	3.65 (3.1)	4.15 (3.8)	4.30 (3.5)	− 1.34 (− 2.29, − 0.39)*	− 1.63 (− 2.48, − 0.77)*
Mindful awareness (PHLMS-Aw)	31.3 (7.7)	29.1 (7.5)	26.4 (8.4)	29.6 (8.1)	28.4 (6.5)	27.1 (8.1)	− 0.76 (− 1.39, 2.91)	− 1.74 (− 0.55, 4.03)
Mindful acceptance (PHLMS-Ac)	35.1 (6.7)	36.0 (6.1)	36.8 (5.9)	33.9 (5.7)	33.5 (5.6)	33.3 (7.3)	1.31 (− 0.45, 3.07)	1.63 (− 0.41, 3.67)
Medication adherence (MARS-A)	37.9 (8.5)	39.5 (9.3)	39.8 (9.7)	38.1 (8.4)	38.4 (8.9)	37.7 (9.0)	0.16 (− 2.16, 2.48)	1.39 (− 0.91, 3.68)

Between group differences are reported as estimated marginal mean difference scores (corrected for baseline values of each measure). Missing data was not imputed and data presented here represent a modified intention to treat analysis, with participants analyzed as randomized but only if they completed the follow up measures. (*) denotes differences in which the 95% confidence interval of group differences does not contain 0. As participants were able to complete postal or online measures, Ns for each analysis have been reported separately

**Table 4:** Within-group comparisons of mean differences from baseline to 6-week and 3-month follow-up questionnaire measures

Postal measures	Baseline vs 6 week Mean difference (95% Confidence interval, effect size)		Baseline vs. 3 month Mean Difference (95% Confidence interval, effect size)	
	Intervention	Control	Intervention	Control
Asthma-related Quality of Life (AQLQ)	0.34* 0.15 to 0.52, *d* 0.46	0.03 − 0.19 to 0.24, *d* 0.04	0.39* 0.18 to 0.59, *d* 0.45	0.11 − 0.13 to 0.36, *d* 0.14
Symptoms subdomain	0.48* 0.24 to 0.73, *d* 0.49	0.02 − 0.28 to 0.32, *d* 0.02	0.43* 0.17 to 0.69, *d* 0.39	0.07 − 0.26 to 0.39, *d* 0.06
Environment subdomain	0.37* 0.10 to 0.64, *d* 0.35	0.21 − 0.06 to 0.50, *d* 0.23	0.62* 0.39 to 0.85, *d* 0.63	0.30 − 0.04 to 0.63, *d* 0.27
Emotions subdomain	0.33* 0.06 to 0.59, *d* 0.31	0.04 –0.32 to 0.41, *d* 0.04	0.41* 0.14 to 0.68, *d* 0.37	0.31 − 0.004 to 0.62, *d* 0.31
Activities subdomain	0.26* 0.07 to 0.45, *d* 0.35	0.01 − 0.23 to 0.26, *d* 0.01	0.25* 0.04 to 0.46, *d* 0.28	0.02 − 0.23 to 0.23, *d* 0.22
Asthma Control (ACQ)	− 0.17* − 0.33 to − 0.01, *d* 0.26	− 0.13 − 0.06 to 0.31, *d* 0.21	− 0.12 − 0.32 to 0.07, *d* 0.14	0.10 − 0.12 to 0.32, *d* 0.14

Online measures	Intervention	Control	Intervention	Control
Anxiety (HADS-A)	0.35 − 0.36 to 1.07, *d* 0.12	1.34* 0.48 to 2.20, *d* 0.49	− 0.66 − 1.36 to 0.04, *d* − 0.24	0.47 − 0.47 to 1.40, *d* 0.15
Depression (HADS-D)	− 0.95* − 1.64 to − 0.27, *d* 0.34	0.73* 0.07 to 1.39, *d* 0.35	− 1.46* − 2.12 to − 0.81, *d* 0.55	0.54 − 0.14 to 1.20, *d* 0.25
Mindful Awareness (PHLMS-Aw)	− 2.20* − 3.92 to − 0.48, *d* 0.32	− 0.42 2.09 to − 1.26, *d* 0.08	− 4.65* − 6.19 to − 3.10, *d* 0.74	− 2.49* − 4.44 to − 0.54, *d* 0.39
Mindful Acceptance (PHLMS-Ac)	0.79 − 0.66 to 2.23, *d* 0.14	0.17 − 0.87 to 1.21, *d* 0.05	0.66 − 0.75 to 2.07, *d* 0.12	0.07 − 1.55 to 1.69, *d* 0.01
Medication Adherence (MARS-A)	− 0.47 − 1.56 to 1.47, *d* 0.01	− 0.07 1.83 to 1.68, *d* 0.01	0.15 − 1.32 to 1.63, *d* 0.03	− 1.14 − 2.96 to 0.68, *d* 0.19

(*) denotes group differences in which the 95% CI does not include 0

#### Asthma Quality of Life Questionnaire (AQLQ)

*6-week follow-up:* Between group comparison (correcting for baseline differences) did not find significantly higher AQLQ scores in the intervention group compared to the control group (Mean Difference [MD] 0.20, 95%CI − 0.06 to 0.46). Within-group analysis from baseline to 6-weeks showed significantly improved AQLQ score in the intervention group, but not in the control group.

*3-month follow-up:* Between group comparisons (correcting for baseline differences) also did not show significantly improved AQLQ scores in the intervention group compared to the control group (MD 0.15, 95%CI − 0.13 to 0.42). As with the 6-week analysis, within-group mean AQLQ score changes from baseline in the intervention group significantly improved, but did not in the control group.

#### Asthma Quality of Life Subscales: Symptoms (AQLQ-S), Environment (AQLQ-En), Emotion (AQLQ-En), Activities (AQLQ-A).

*6-week follow-up:* Between group comparisons (correcting for baseline differences) did not show significant improvements in the intervention group in subdomain scores of symptoms (MD 0.25, 95%CI − 0.08 to 0.59), environment (MD 0.10, 95%CI − 0.24 to 0.45), emotions (MD 0.08, 95%CI − 0.32 to 0.49). and activities (MD 0.20, 95%CI − 0.09 to 0.48).

Within group comparisons from baseline to 6-weeks showed the intervention group improved significantly in all subdomains, while the control group did not significantly improve in any subdomains.

*3-month follow-up:* Between group comparisons (correcting for baseline) did not show significant improvements subdomain scores of symptoms (MD 0.15, 95%CI − 0.21 to 0.51), environment (MD 0.26, 95%CI − 0.08 to 0.60), activities (MD 0.20, 95%CI − 0.11 to 0.51) and emotions (MD − 0.11, 95%CI − 0.45 to 0.23) in the intervention group compared to the control group.

Within group comparisons from baseline to 3-months showed the intervention group improved significantly in all subdomains while the control group did not significantly improve in any subdomains.

#### Asthma Control Questionnaire (ACQ)

*6-week follow-up:* Between group comparisons (correcting for baseline) showed asthma control did not significantly improve in the intervention group above the control group (MD − 0.26, 95%CI − 0.49 to − 0.03; with a lower ACQ score indicating improved control). Within group comparisons showed that the intervention group had significantly improved asthma control vs. baseline while asthma control in the control group did not significantly change.

*3-month follow-up:* Between-group comparisons (correcting for baseline) did not show significant improvement in ACQ score in the intervention group above the control group (MD − 0.17, 95%CI − 0.44 to 0.10). Within-group comparison from baseline to 3-months did not show significant improvement in the intervention group, nor and did not show significant worsening in the control group.

### Secondary analysis: anxiety, depression, mindfulness and medication adherence

Additional outcomes of anxiety (HADS-A), depression (HADS-D), mindfulness (PHLMS-Aw and PHLMS-Acc) and medication adherence (MARS-A) were examined using group comparisons at follow-up (correcting for baseline differences in each measure). Data from both 6-week and 3-month follow-up is reported in full in Table 3, and within group comparisons are reported in Table 4.

At 6-week follow-up, the intervention group had significantly lower depression scores at follow-up compared to the control group (MD − 1.34, 95%CI − 2.29 to − 0.39). The intervention group did not show significantly different scores for anxiety, mindful awareness, mindful acceptance or medication adherence compared to the control group.

The same pattern was observed at 3-month follow-up. In comparison with the control group, the intervention group had significantly lower depression scores (MD − 1.63, 95%CI − 2.48 to − 0.77), but not significantly different scores in anxiety, mindful awareness, mindful acceptance, or adherence.

Within-groups analysis showed that after 6-weeks the intervention group had significantly improved depression and mindful awareness scores, while anxiety, mindful acceptance and medication adherence did not significantly change. After 6-weeks the control group had higher depression scores and anxiety scores but there was no significant change in other measures.

Similarly, after 3 months the intervention group had significantly improved depression and mindful awareness scores than at baseline, with no significant change in anxiety, mindful acceptance or medication adherence. The control group had significantly lower mindful awareness scores but no significant change in other measures.

### Exploratory analysis

#### Comparing ‘engaged’ participants in the intervention group versus control

Exploratory analysis compared asthma quality of life in ‘engaged’ participants in the intervention group i.e. those who accessed the intervention at least once (N = 65) vs. control participants. Between groups comparisons (correcting for baseline differences) showed no significant difference between 6-week AQLQ scores in the engaged group compared to control (MD 0.27, 95%CI − 0.01 to 0.54), and significantly better ACQ scores in the engaged group (MD 0.37, 95%CI 0.14 to 0.59). At 3-months there was no significant difference in asthma quality of life (MD 0.20, 95%CI − 0.09 to 0.49) and asthma control (MD − 0.24, 95%CI − 0.52 to 0.04) between engaged and control participants.

#### Group differences in minimal clinically important change (MCID)

**Fig. 2:**
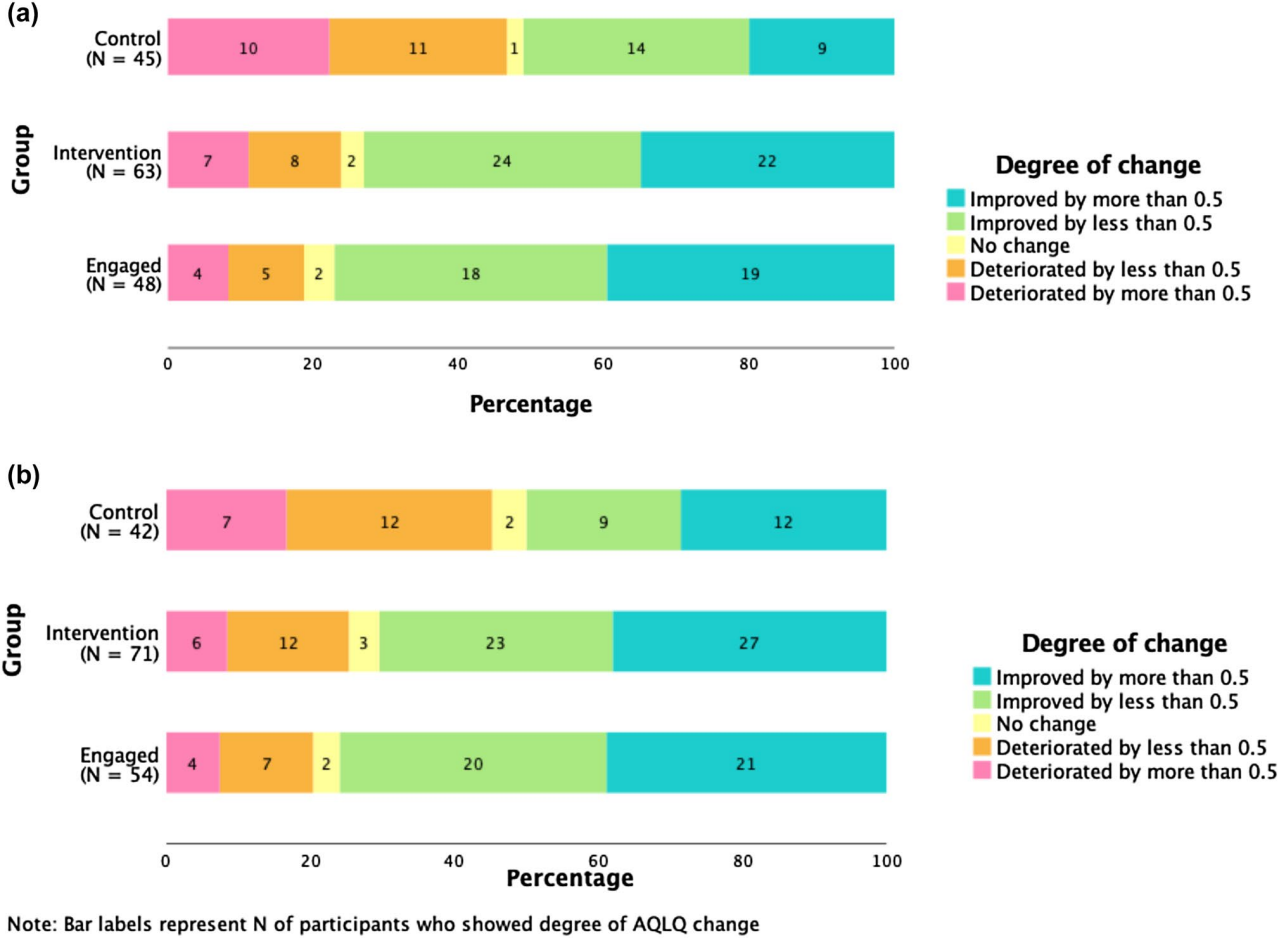
(**a**) Proportion of participants who demonstrated a change in primary endpoint at 6 weeks, relevant to minimum clinically important difference (MCID). (**b**) Proportion of participants who demonstrated a change in primary endpoint at 3 months, relevant to minimum clinically important difference (MCID)

Individual subject changes in AQLQ scores from baseline were assessed according to the achievement of MCID (0.5). The proportion of participants who had greater and less than MCID change in AQLQ is presented in Fig. 2. A greater proportion of participants in the intervention group (6 weeks: 35%, 3 months: 38%) showed an MCID-relevant improvement in quality of life than in the control group (6 weeks: 20%, 3 months: 29%), and a higher percentage of control group showed a relevant decrease in quality of life (6 weeks: 22%, 3 months: 17%) than intervention (6 weeks: 11%, 3 months: 9%).

Using recommended analysis for the primary endpoint (Guyatt et al., 1998), the number needed to treat (NNT) for one subject randomized to active arm to achieve a relevant improvement in quality of life above control was 7.27 (see Table 5).

**Table 5:** Number need to treat at 3-month follow-up (all patients who completed baseline and 3-month data)

		Intervention group		
		Improved > MCID	Unchanged	Deteriorated > MCID
Control group	Improved > MCID	0.11	0.15	0.02
	Unchanged	0.21	0.3	0.05
	Deteriorated > MCID	0.06	0.09	0.01
		Proportion who received a benefit:		0.14
		NNT:		7.27

Logistic regression analysis was used to compare the proportion of participants achieving an improvement greater than MCID after controlling for baseline AQLQ score. At 3 months, those in the intervention group were non-significantly more likely to achieve a relevant improvement (OR 1.23, 95% CI 0.46 to 3.28).

## Discussion

This pragmatic, randomized feasibility trial shows that the digital mindfulness intervention ‘Headspace’ is relevant and acceptable to at least a proportion of people with asthma, with the potential to benefit patients, so merits a fully-powered confirmatory RCT. Recruitment targets were achieved, randomization procedures were successful, and invitation response and retention rates were comparable with previous randomized controlled trials of digital interventions in similar populations (McLean et al., 2016). Our sample ranged from 18 to 90 years old, demonstrating the potential utility of digital interventions to reach a broad patient demographic. Our questionnaire response rates were in line with previous relevant research (Ainsworth et al., 2019a, 2019b; Morrison et al., 2016) although it should be noted that using both internet and postal measures meant that some patients did not complete all measures (providing valuable information for a future trial).

The remote nature of our trial procedures (with recruitment, enrolment, randomization, intervention and follow-up all occurring online or via post), was efficient and effective as a design. While recruitment rates per practice were slightly lower than in other similar studies (e.g. Ainsworth et al., 2019a, 2019b), the efficient use of resources in remote designs meant we were still able to recruit successfully. Recruitment could be further improved by incorporating additional methods that lend themselves to remote trials, such as social media. There are some drawbacks to remote designs; including the inability to examine such physiological measures as lung function and a reliance on patients to accurately report symptoms and screening criteria. Careful consideration is needed to ensure procedures and interventions do not place patients at risk during remote studies.

Our primary outcomes of interest—asthma quality of life and asthma control—both demonstrated substantial improvement in the intervention group above baseline values, and consistent trends to improvements over the control group. A greater proportion of participants in the intervention group demonstrated an improvement of the minimally important clinical difference in asthma-specific quality of life, with a low number needed to treat of below 5 for a patient to experience a relevant improvement in asthma control. We also observed indications of more positive anxiety and depression scores at follow-up in comparison to the control group.

Although this feasibility study was not powered to evaluate differences between groups, our study consistently found promising indications of improved outcomes for those in the intervention group compared to the control group across almost all patient-reported questionnaires, indicating the potential benefit of the intervention. Importantly, we did not find evidence of change in medication adherence to explain the improvements observed, consistent with the notion that MBTs may act as an adjunct intervention to standard pharmacological treatments to improve quality of life.

One of the strengths of the study was that patient engagement with the Headspace app exceeded other similar digital interventions in primary care patients with asthma (McLean et al., 2016). This suggests that this digital mindfulness intervention may be acceptable and accessible for many people with asthma. However, some participants did not use the intervention at all, suggesting that intervention reach could still be improved, and that this intervention may not be acceptable to all. Of those who did engage, the range of usage was large: some used it once or twice during the entire study whilst others used it several times a day. This is in line with cutting edge theories of digital behavior change interventions: accessible interventions should be flexibly designed to allow for different usage patterns to suit individuals who may engage with behaviors in a variety of different ways that suit them (Ainsworth et al., 2017). In this study, we did not advise how frequently participants should use Headspace, nor if they should access specific components. However, detailed usage data was regarding specific Headspace practices that were preferred by individuals (e.g. ‘Managing Anxiety’ and ‘Stress’) which will inform the development of ‘asthma-specific’ content to improve acceptability and effectiveness in a future trial. For example, initial information provision of potential asthma-related benefits of practicing mindfulness may have led to fewer non-engagers in our study. We also note that the exploratory nature of the feasibility study meant that we did not integrate our data-gathering platform (Lifeguide) with the intervention platform (Headspace) and therefore required participants to sign up to each individually streamlining these for a full trial would likely result in even more effective engagement. We also suggest using theory- and person-based approaches to further maximize acceptability and effectiveness (Yardley et al., 2015). In line with MRC guidance for intervention development and evaluation (Duncan et al., 2020) we have conducted a detailed embedded process that provides further detailed guidance on intervention optimization (Stanescu et al., 2021).

As well as the encouraging results, this study had a sufficient sample size to support confidence in the exploratory findings. While the 2:1 randomization process allows increased variability in the control group, it generated detailed intervention usage data that will inform a further full trial. The online nature of the study meant that study recruitment was particularly cost-effective and facilitated rigid study procedures, with very little possibility for researcher bias or protocol deviation).

There are several limitations to this study which must be acknowledged. Firstly, baseline comparisons indicated that those randomized to the intervention group tended towards impaired quality of life compared to the control group at baseline. Although our primary analysis controlled for these different baseline values, participants in the control group may have experienced a ceiling effect, and consequently had a reduced magnitude of improvement.

Although our findings suggested benefits of the intervention at 6-weeks and 3-months, this study does not explore long-term evidence of benefit (e.g. for over a year). While it is possible that the benefits of mindfulness practice accrue over time, it may be that initial levels of engagement with the digital intervention ‘drop off’ as good habits formed by participants subside. Indeed, the inconsistent and heterogenous nature of asthma symptoms mean that other digital interventions have included specific content to remind users to re-engage when symptoms appear (Ainsworth et al., 2019a) and this should be included in a larger randomized controlled trial.

A complex behavioral intervention such as mindfulness means that participants are not blind to their group allocation. However, psychological benefits to receiving a treatment are, in the case of mindfulness, fundamental treatment components that should be included in evaluation (Ainsworth et al., 2019b) and therefore we consider our pragmatic feasibility trial an effective design, especially given the remote nature of the study (i.e. researcher blinding could not be an issue). Similarly, ‘standard asthma management’ that participants in the control group received may be impacted by enrolment in the study—an issue which should be explored in future research.

Of more concern is the consideration of ‘reach’. Around 4% of eligible patients enrolled in the study, and it is possible that patients with the most impaired quality of life and asthma control (who are likely to benefit most from adjunct therapies) may not be willing (or financially able) to sign up to digital interventions, particularly treatments such as mindfulness. While mindfulness is increasingly common in the public sphere (and therefore increasingly acceptable; (Kachan et al., 2017)), and internet access is more widespread, care must be taken not to entrench digital inequality (Hargittai et al., 2018). Similarly, our sample could have been more diverse; for example, we recruited relatively few non-white participants. Therefore, any further research must use evidence, theory- and person-based approaches to ensure that a full trial and subsequent dissemination is accessible for as inclusive and diverse a demographic as possible.

The remote nature of the study also meant that we were unable to measure objective, physiological markers of asthma, such as lung function and health-resource use. Although evidence suggests that subjective self-report is a more accurate predictor of quality of life (Janssens et al., 2012), such measures should not be overlooked in order to understand the mechanisms by which the observed improvements in asthma control and asthma quality of life occurred. Understanding the mechanisms of psychological and behavioral treatments is important to determine whether the benefits of such treatments are ‘non-specific’ (see Ainsworth et al., 2019b) or target specific psychological mechanisms that may be dysfunctional in patient groups. Mindfulness, which advocates a non-judgemental awareness of thoughts and feelings, may lead to better asthma outcomes through improved symptom perception—which has previously been demonstrated to be a better predictor of quality of life for people with asthma than objective lung function impairment (Janssens et al., 2012). Similarly, mindfulness may improve illness perceptions (beliefs and emotional responses to their condition)—which subsequently effect a range of asthma outcomes including disease management (Kaptein et al., 2010). Although we found potential benefits in disease-specific quality of life, it may be that mindfulness practice leads to a general improvement in mood that, while not specific to asthma, ultimately improves patient quality of life measures that are specifically relevant for asthma evaluation. Further research should complement pragmatic trial studies through experimental studies that detect the degree to which subjective improvement is associated with changes in lung function alongside changes in non-disease-specific subjective outcomes.

## Conclusion

This study demonstrated the feasibility of a digital mindfulness intervention for people with asthma in primary care, suggesting benefits for asthma control and quality of life, anxiety and depression. The intervention was acceptable to patients, although engagement levels varied across the sample. With appropriate modification of trial procedures, these data support the feasibility of a confirmatory randomized-controlled trial.

## Supplementary Information

Below is the link to the electronic supplementary material.

Supplementary file1 (PDF 185 KB)

Supplementary file2 (PDF 151 KB)

## Data Availability

The analysis scripts used during the current study are available in the FIGSHARE repository: https://doi.org/10.6084/m9.figshare.11956761.v1. The data is available in line with the consent collected from participants; by request.

## Abbreviations

ACQ: Asthma Control Questionnaire
AQLQ: Asthma Quality of Life Questionnaire
GP: General practitioner
HADS: Hospital Anxiety and Depression Scale
MARS-A: Medical Adherence Report Scale
MBIs: Mindfulness-based interventions
MBCT: Mindfulness-based cognitive therapy
MBSR: Mindfulness-based stress reduction
MCID: Minimum clinically important difference
MD: Mean difference
NIHR: National Institute of Health Research
NNT: Number needed to treat
OR: Odds ratio
PHLMS: Philadelphia Mindfulness Scale
RCT: Randomized controlled trial
