# Supplementary material for: A feasibility trial of a digital mindfulness-based intervention to improve asthma-related quality of life for primary care patients with asthma
Source: J Behav Med. 2021 Aug 27;45(1):133–47. doi: 10.1007/s10865-021-00249-3 (PMC8818629; doi:10.1007/s10865-021-00249-3)
Supplement: Supplementary file 2 — Supplementary file2 (PDF 151 KB) [file 10865_2021_249_MOESM2_ESM.pdf]

Supplementary File 2. Baseline participant demographic information for participants who withdrew from the study or were lost to follow up and did not complete the primary endpoint (LTFU) at 6 weeks and 3 months.

Measure	**Final sample (N = 144)**	**Withdrawn (N = 14)**	**LTFU at 6 weeks (N = 20)**	**LTFU at 3 months (N = 11)**
Age: M (SD)*	51.1 (14.7)	58.2 (10.9)	43.2 (14.0)	38.3 (14.4)
Ethnicity (%)	White (97%), Indian (3%)	White (100%)	White (100%)	White (81%), Chinese (9%), other (1%)*
Education (%)	School (22%), Degree/Diploma (57%), Postgraduate (20%), Other (1%)	School (40%), Diploma (20%), Degree (20%), Other (20%)	School (25%), Degree/Diploma (45%) Postgraduate (30%).	School (27%), Degree/Diploma (55%), Postgraduate (18%).
Weekly internet use: M hours (SD)	17.2 (14.3)	15.1 (7.5)	16.7 (14.4)	26.3 (26.4)
Meditation experience (%)	Not heard of it/don't know about it (14%), never tried it (30%), tried other types of meditation (23%), tried mindfulness (25%), regularly practice mindfulness (7%).	Not heard of it/don't know about it (20%), never tried it (60%), tried other types of meditation (20%).	Not heard of it/don't know about it (10%), never tried it (35%), tried other types of meditation (30%), tried mindfulness (20%), regularly practice mindfulness (5%).	Not heard of it / don't know about it (18%), never tried it (54%), tried other types of meditation (9%), tried mindfulness (9%), regularly practice mindfulness (9%).
Years since diagnosis	26.1 (15.9)	19.2 (19.2)	29.8 (18.1)	23.4 (19.1)
Asthma-related Quality of Life (AQLQ)	5.44 (1.0)	5.72 (0.9)	5.27 (1.1)	5.24 (1.2)
Symptoms subdomain	5.24 (1.2)	5.45 (1.0)	5.11 (1.2)	4.87 (1.4)
Environment subdomain	4.97 (1.3)	5.30 (1.3)	4.78 (1.3)	4.80 (1.5)
Emotions subdomain	5.44 (1.3)	5.80 (1.0)	5.22 (1.3)	5.33 (1.3)
Activities subdomain	5.91 (1.0)	5.91 (1.0)	5.80 (1.2)	5.84 (1.0)
Asthma Control (ACQ)	1.14 (0.9)	1.02 (0.7)	1.25 (0.9)	1.37 (1.0)
Anxiety (HADS-A)	7.72 (4.3)	5.79 (3.8)	8.58 (3.7)	8.50 (4.3)
Depression (HADS-D)	4.34 (3.7)	4.00 (3.7)	4.97 (3.7)	3.93 (3.1)
Mindful Awareness (PHLMS-Aw)	30.7 (7.9)	27.6 (6.6)	30.1 (7.3)	32.1 (7.3)
Mindful Acceptance (PHLMS-Ac)	34.7 (6.4)	33.2 (3.6)	35.3 (6.5)	34.0 (7.5)
Medication Adherence (MARS-A)	37.9 (8.5)	39.4 (9.0)	38.2 (8.8)	35.8 (7.9)

Note(s): Some demographic questionnaires were not compulsory and the data reported is from all available participants. ACQ (lower scores equate to better control); AQLQ (higher scores equate to greater impairment); HADS (higher scores equate to more anxiety). PHLMS (two subscales of awareness and attention, in which higher scores equate to more mindfulness); MARS-A (higher scores equate to better adherence). *Chi-square test could not be conducted due to insufficient cases in cells.
